# Supplementary material for: The Over-Expression of Two R2R3-MYB Genes, PdMYB2R089 and PdMYB2R151, Increases the Drought-Resistant Capacity of Transgenic Arabidopsis
Source: Int J Mol Sci. 2023 Aug 30;24(17):13466. doi: 10.3390/ijms241713466 (PMC10487491; doi:10.3390/ijms241713466)
Supplement: Supplementary file 1 [file ijms-24-13466-s001.zip › ijms-2521257-supplementary-final/ijms-2521257-supplementary.pdf]

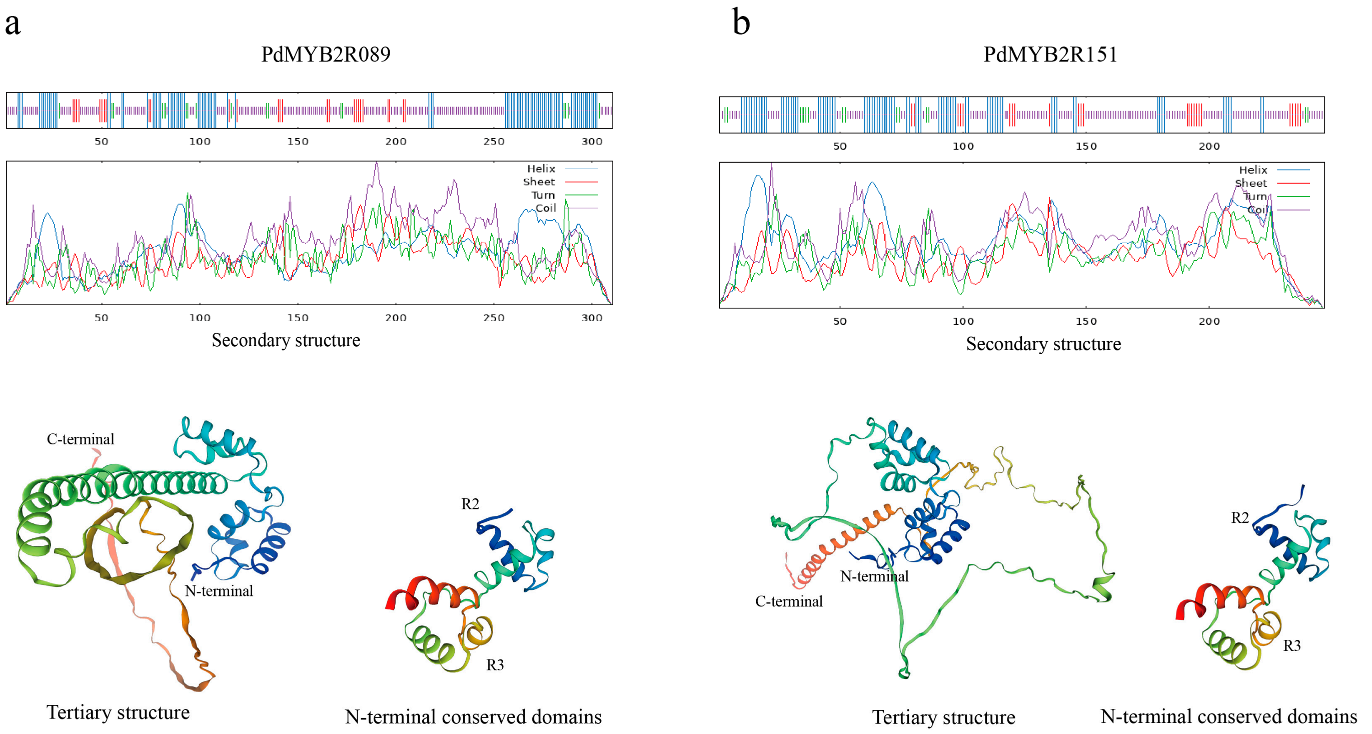

Supplementary Figure S1: The secondary structure and tertiary structure of PdMYB2R089/151 protein. (a) shows secondary structure and tertiary structure of PdMYB2R089 protein. (b) shows secondary structure and tertiary structure of PdMYB2R151 protein.

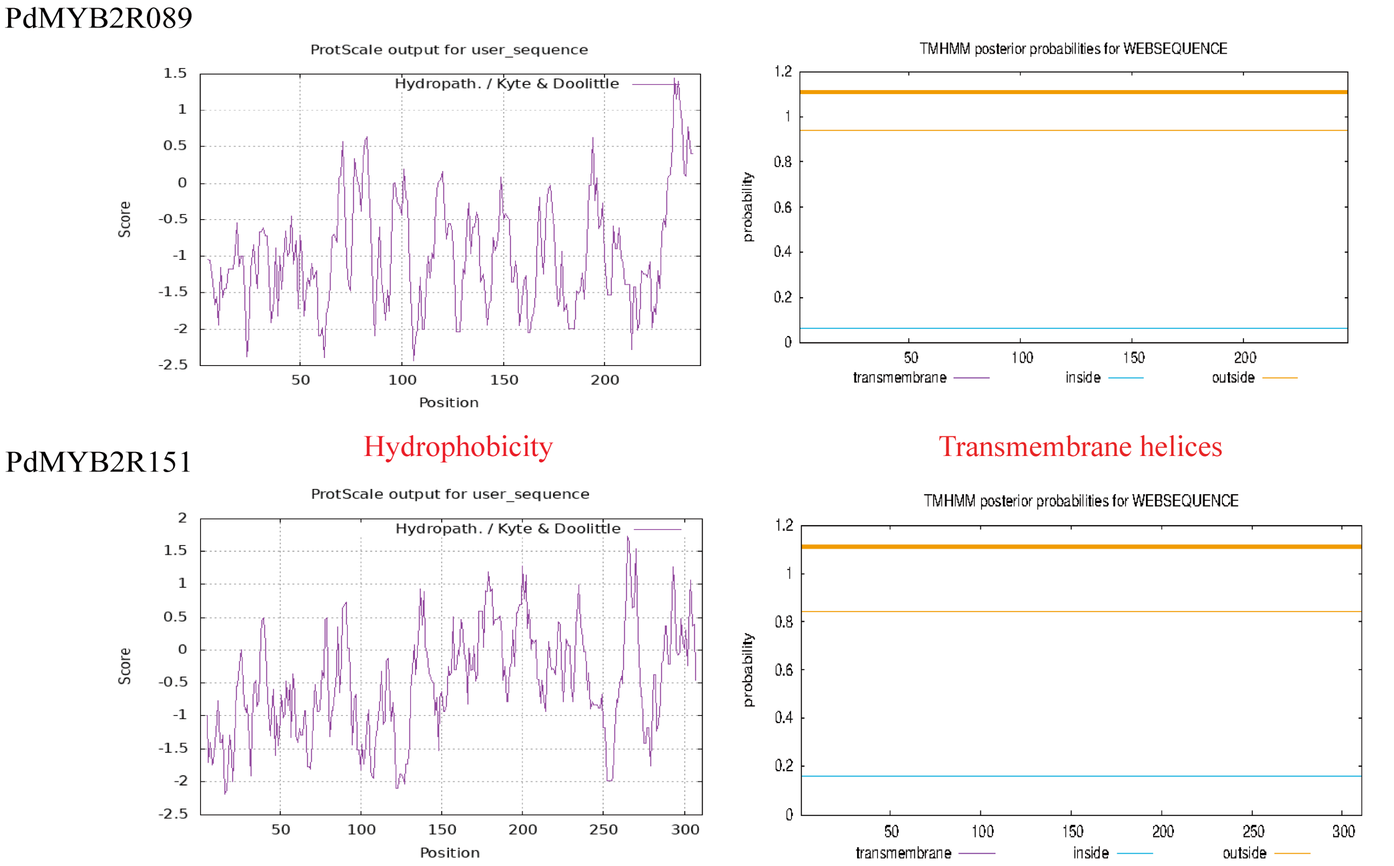

Supplementary Figure S2: The hydrophobicity and transmembrane domain of PdMYB2R089/151 protein;

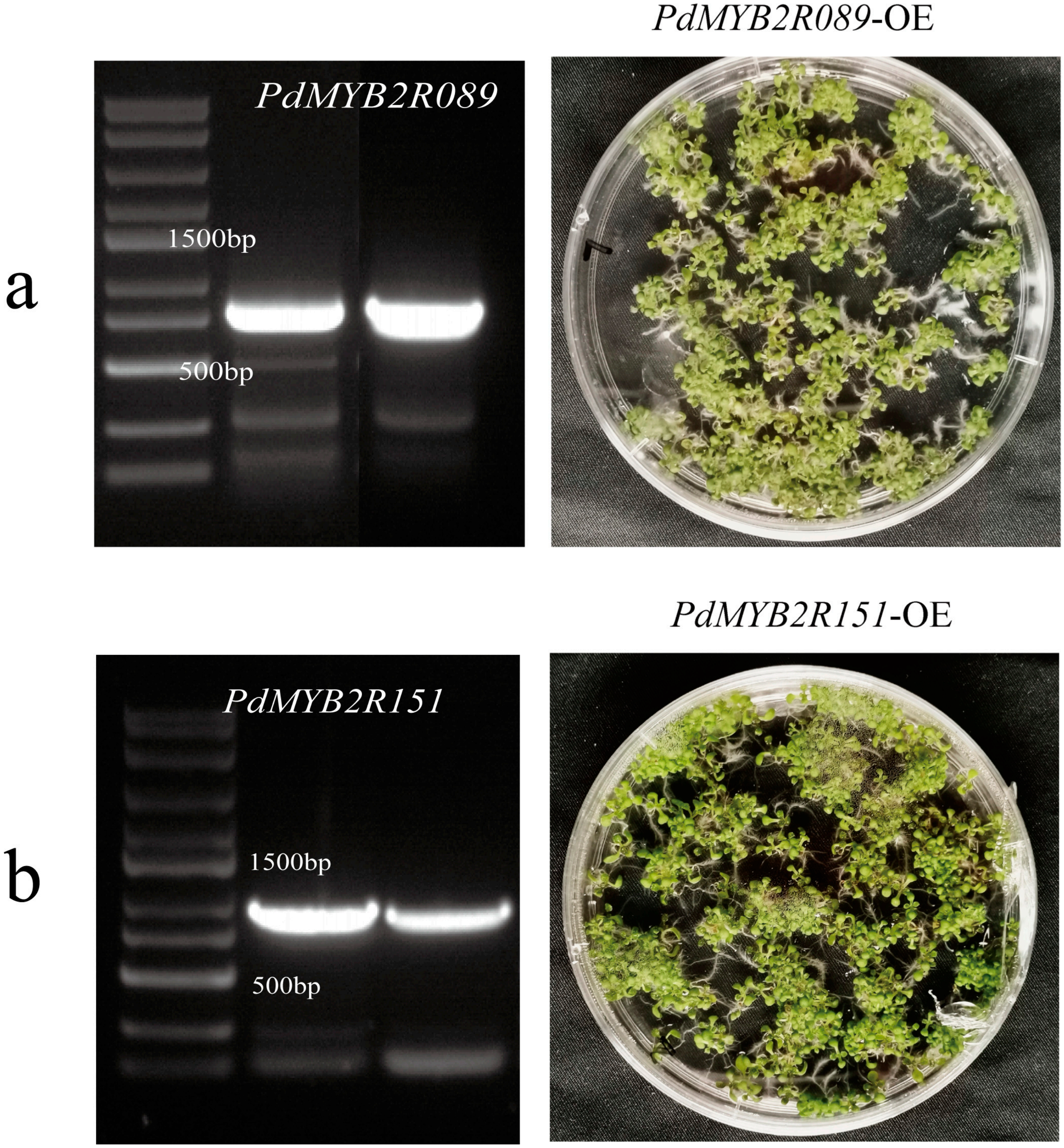

Supplementary Figure S3: PCR detection of overexpressed transgenic plants and homozygous transgenic plants. (a) shows PCR detection and homozygous *PdMYB2R089*-OE transgenic plants. (b) shows PCR detection and homozygous *PdMYB2R151*-OE transgenic plants.

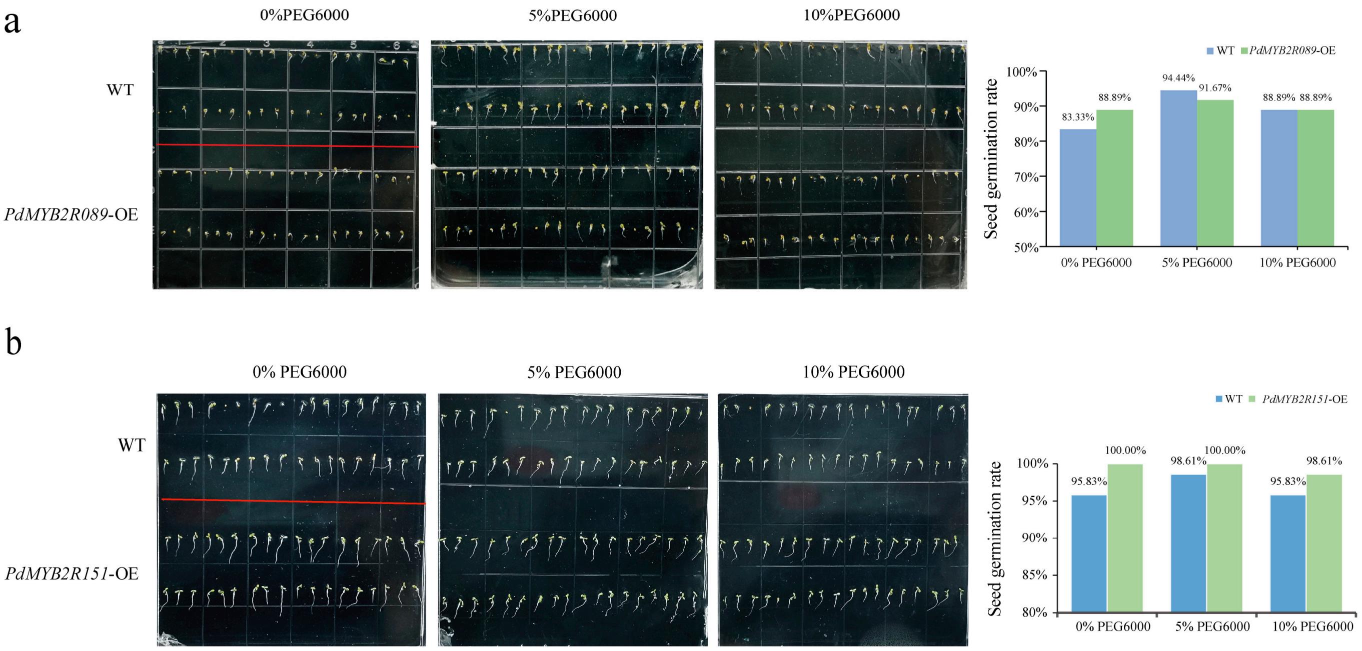

Supplementary Figure S4: Seed germination of WT and transgenic plants under different PEG6000 concentration gradients. (a) shows seed germination of WT and *PdMYB2R089*-OE plants under drought stress. (a) shows seed germination of WT and *PdMYB2R089*-OE plants under drought stress.

Supplementary Table S1: Physicochemical properties analysis of PdMYB2R089/151 protein;

Supplementary Table S2: Primer information is required for vector construction.

Supplementary Table S3: Protein information for protein-protein interaction prediction.
