# Supplementary figures and images for: The Over-Expression of Two R2R3-MYB Genes, PdMYB2R089 and PdMYB2R151, Increases the Drought-Resistant Capacity of Transgenic Arabidopsis
Source: Int J Mol Sci. 2023 Aug 30;24(17):13466. doi: 10.3390/ijms241713466 (PMC10487491; doi:10.3390/ijms241713466)

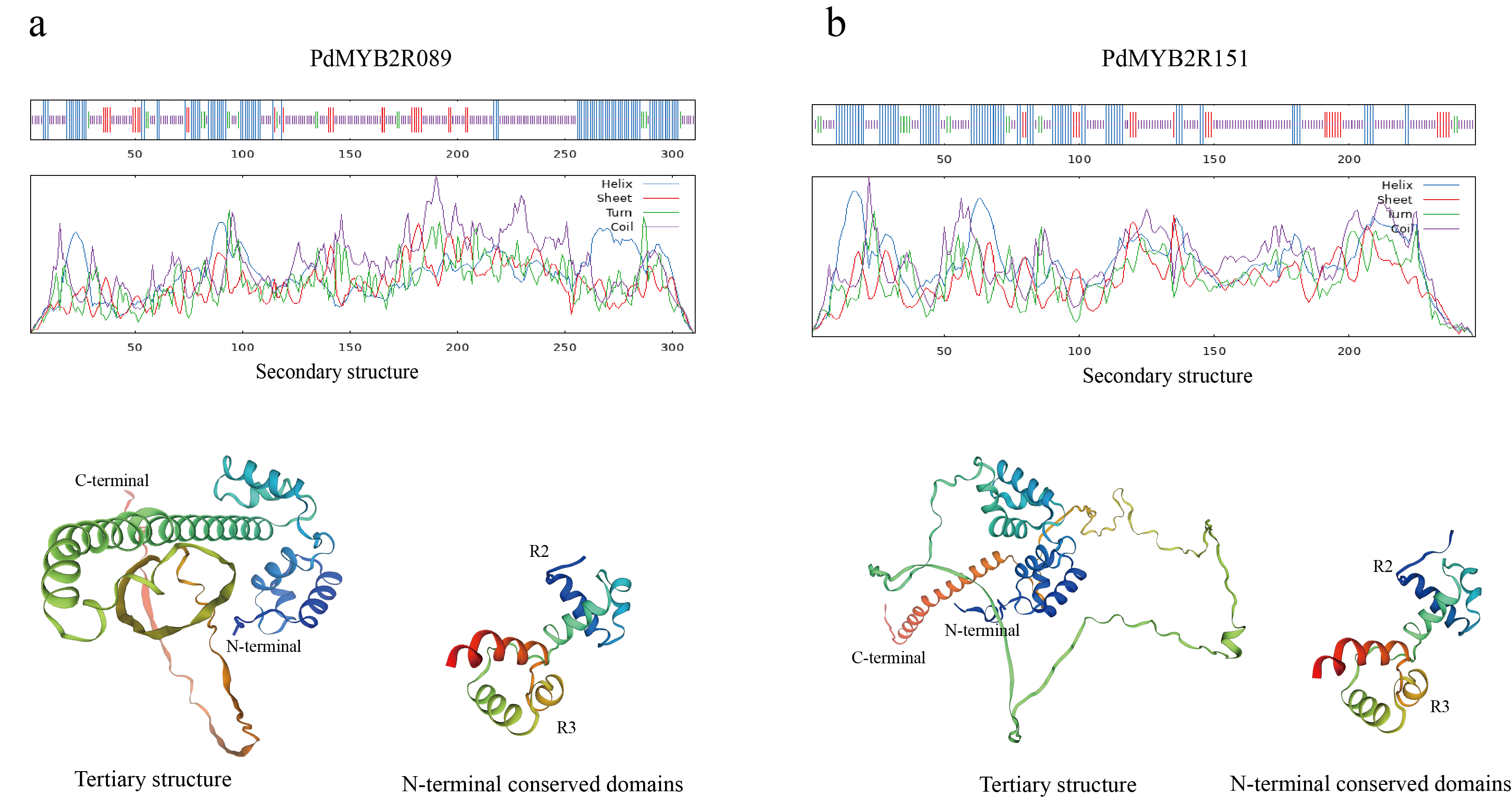

Supplement: Supplementary file 1 [file ijms-24-13466-s001.zip › ijms-2521257-supplementary-final/Supplementary Figure S1.png]

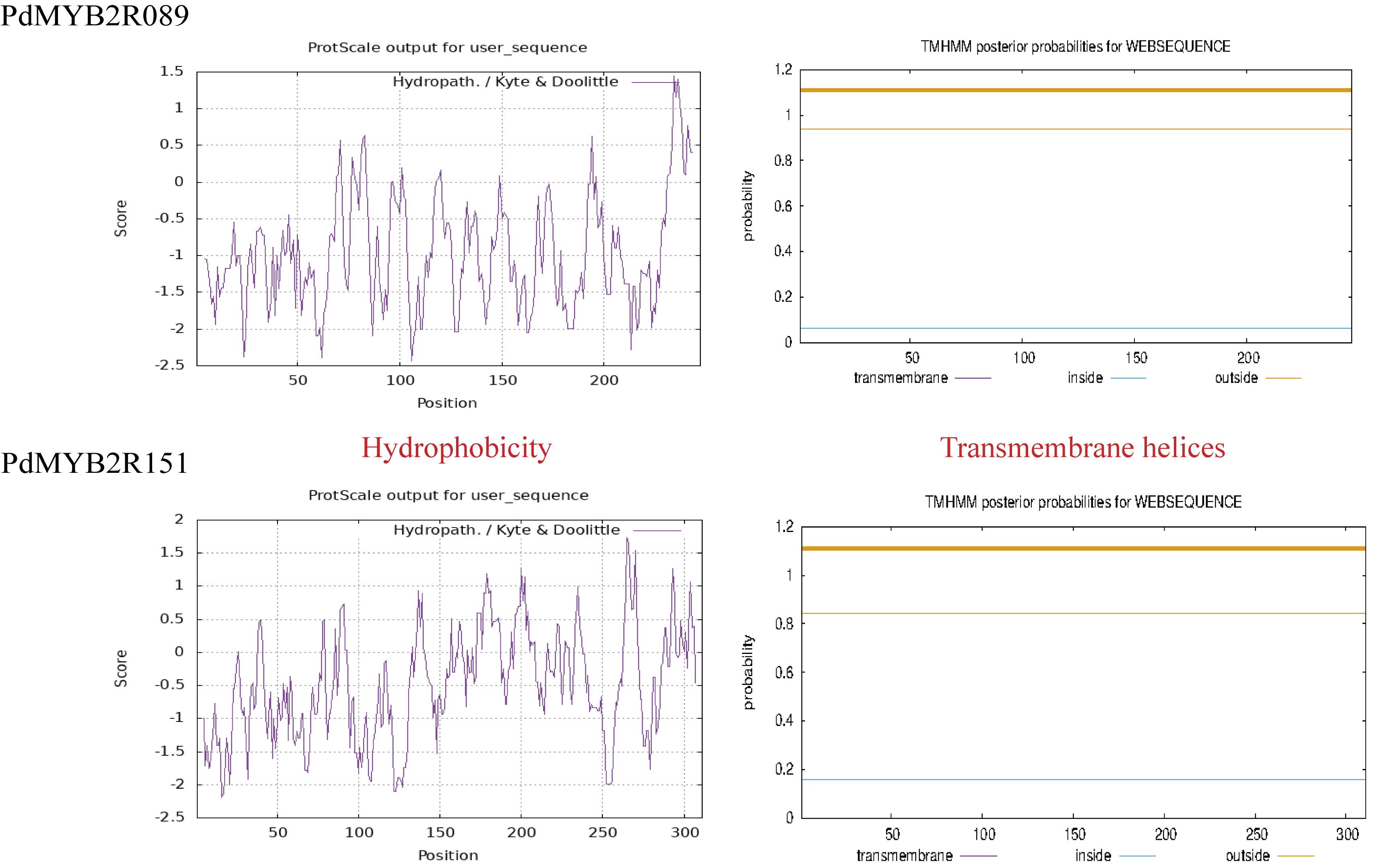

Supplement: Supplementary file 1 [file ijms-24-13466-s001.zip › ijms-2521257-supplementary-final/Supplementary Figure S2.png]

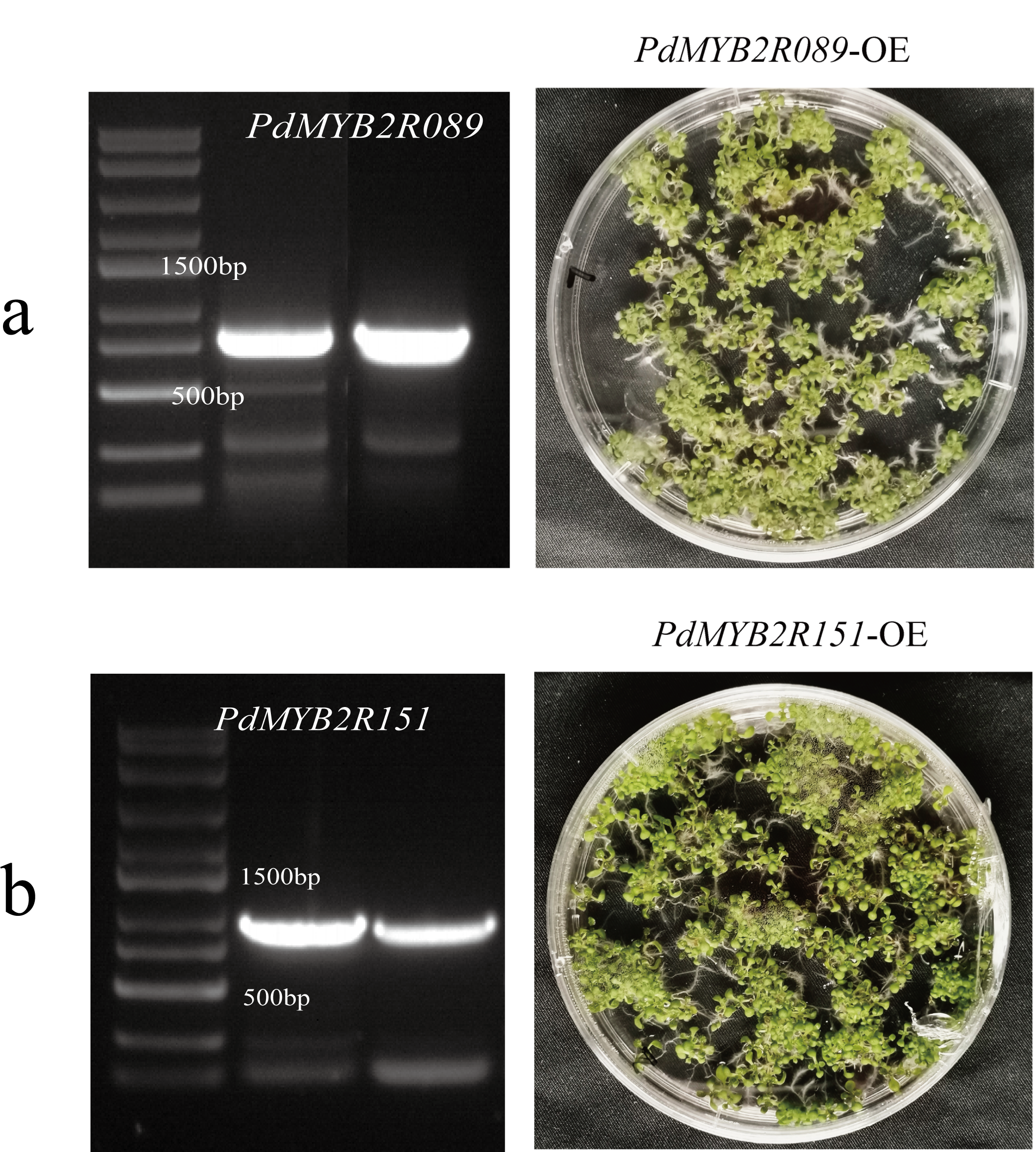

Supplement: Supplementary file 1 [file ijms-24-13466-s001.zip › ijms-2521257-supplementary-final/Supplementary Figure S3.png]

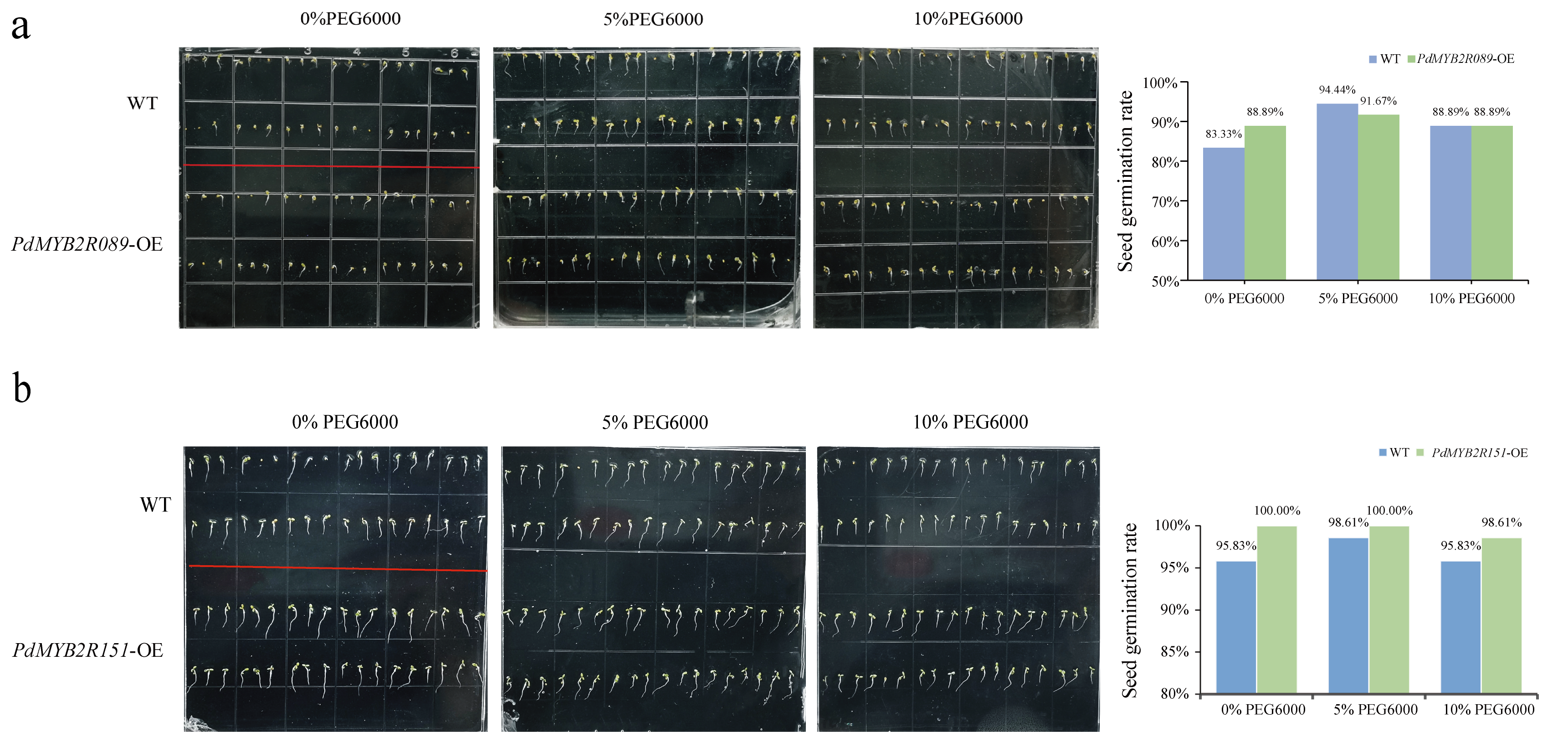

Supplement: Supplementary file 1 [file ijms-24-13466-s001.zip › ijms-2521257-supplementary-final/Supplementary Figure S4.png]
